# A Methodology for the Enzymatic Isolation of Embryonic Hypothalamus Tissue and Its Acute or Post-Culture Analysis by Multiplex Hybridisation Chain Reaction

**DOI:** 10.21769/BioProtoc.4898

**Published:** 2023-12-05

**Authors:** Kavitha Chinnaiya, Marysia Placzek

**Affiliations:** School of Biosciences, University of Sheffield, Sheffield, UK

**Keywords:** Embryonic hypothalamus dissection, Enzymatic tissue dissection, Explant culture, Multiplex hybridisation chain reaction (HCR), Stripping and re-probing protocol for HCR

## Abstract

The hypothalamus is an evolutionarily ancient part of the vertebrate ventral forebrain that integrates the dialogue between environment, peripheral body, and brain to centrally govern an array of physiologies and behaviours. Characterizing the mechanisms that control hypothalamic development illuminates both hypothalamic organization and function. Critical to the ability to unravel such mechanisms is the skill to isolate hypothalamic tissue, enabling both its acute analysis and its analysis after explant and culture. Tissue explants, in which cells develop in a manner analogous to their in vivo counterparts, are a highly effective tool to investigate the extrinsic signals and tissue-intrinsic self-organising features that drive hypothalamic development. The hypothalamus, however, is induced and patterned at neural tube stages of development, when the tissue is difficult to isolate, and its resident cells complex to define. No single molecular marker distinguishes early hypothalamic progenitor subsets from other cell types in the neural tube, and so their accurate dissection requires the simultaneous analysis of multiple proteins or mRNAs, techniques that were previously limited by antibody availability or were arduous to perform. Here, we overcome these challenges. We describe methodologies to precisely isolate early hypothalamic tissue from the embryonic chick at three distinct patterning stages and to culture hypothalamic explants in three-dimensional gels. We then describe optimised protocols for the analysis of embryos, isolated embryonic tissue, or cultured hypothalamic explants by multiplex hybridisation chain reaction. These methods can be applied to other vertebrates, including mouse, and to other tissue types.

Key features

• Detailed protocols for enzymatic isolation of embryonic chick hypothalamus at three patterning stages; methods can be extended to other vertebrates and tissues.

• Brief methodologies for three-dimensional culture of hypothalamic tissue explants.

• Optimised protocols for multiplex hybridisation chain reaction for analysis of embryos, isolated embryonic tissues, or explants.

**Graphical overview**


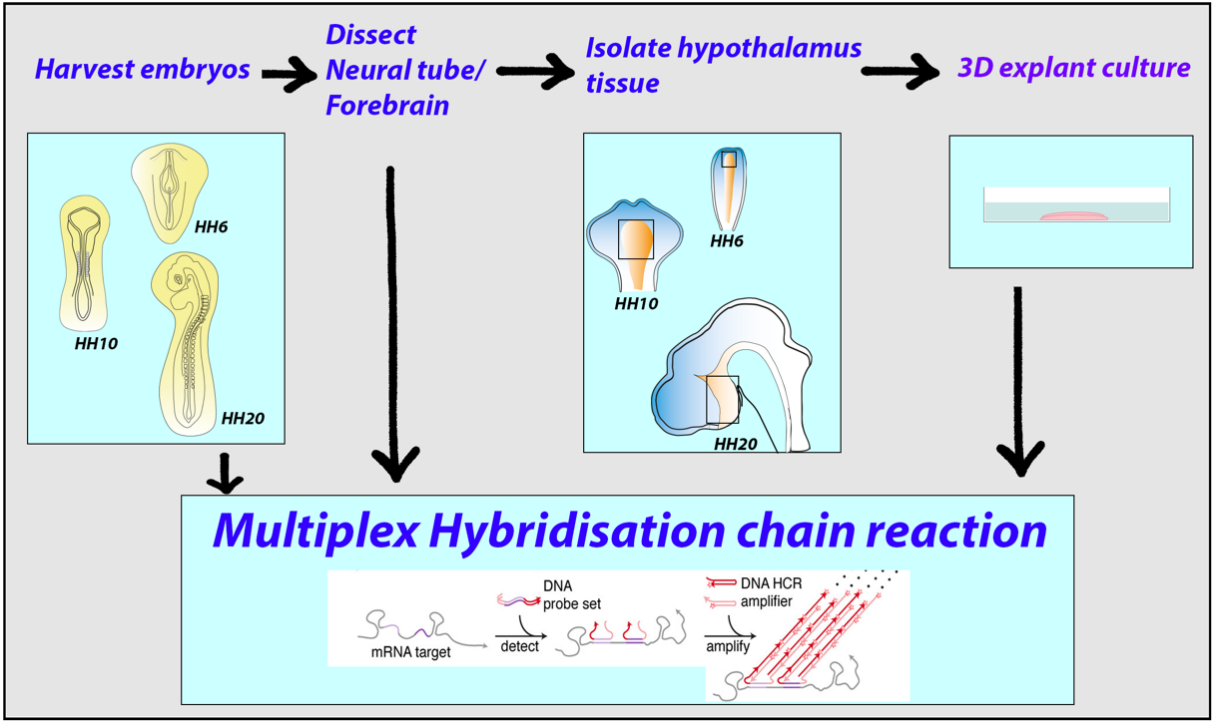


## Background

In the developing embryo, dynamic cellular and molecular processes direct early tissue patterning and subsequent organogenesis. These events have been deciphered through multiple experimental approaches, but one of the most indispensable tools in such investigations is the use of tissue or organ explants. While organs—including the central nervous system—can be isolated manually (Moore and Kennedy, 2008; Morrison et al., 2021), tissue isolation at patterning stages requires an enzymatic treatment. A variety of different enzymes can be used, including Collagenase, Trypsin, Dispase I, or Dispase II (Davies, 2010; Lowery et al., 2012; Morales et al., 2016; Shirahama et al., 2019; Ye et al., 2022), amongst which Dispase I is particularly effective (Placzek et al., 1990). This neutral protease can separate intact tissue layers, preserving the viability of each layer, and has frequently been used to isolate the posterior neural tube (Placzek et al., 1990; Yamada et al., 1993; Ericson et al., 1997; Tozer et al., 2013). Explants from isolated posterior neural tube regions, or adjacent tissues, have enabled an exquisite dissection of the cellular interactions and signalling events that orchestrate spinal cord patterning. In combination with post-hoc analysis through immunohistochemistry or in situ hybridisation, their use has helped to reveal how distinct progenitor subtypes are established along the dorso–ventral axis of the prospective spinal cord in response to dynamic morphogen gradients (Grega, 1984; Placzek et al., 1990; Yamada et al., 1993; Ericson et al., 1997; Tozer et al., 2013; Morales et al., 2016; Placzek and Briscoe, 2018).

The anterior neural tube that will give rise to the brain is more difficult to isolate due to its small size and dynamic morphogenesis. Nonetheless, where anterior neural tube explants have been used, they, too, have provided new insights into the tissue, cell, and molecular interactions that mediate brain patterning (Ericson et al., 1995; Guinazu et al., 2007; Placzek and Briscoe, 2018; Shirahama et al., 2019). In particular, their use has significantly improved our understanding of the development of the hypothalamus (Dale et al., 1997; Manning et al., 2006; Kim et al., 2022; Chinnaiya et al., 2023). This intricate region of the ventral forebrain regulates physiological processes that maintain homeostasis and orchestrate complex behaviours. The enzymatic isolation of entire embryonic chick hypothalamic tissues at different patterning stages, and their acute analysis through scRNA-seq, has generated a comprehensive roadmap of hypothalamic development, from induction to patterning and to neurogenesis, and revealed many previously uncharacterized candidate regulators of hypothalamic patterning (Kim et al., 2022). A powerful new development has been the use of multiplex hybridisation chain reaction (HCR) (Choi et al., 2018) in the analysis of wholemount and isolated hypothalamic tissues or hypothalamic tissue explants (Kim et al., 2022; Chinnaiya et al., 2023), for detecting and visualising specific DNA or RNA sequences in a sample. In the protocol we describe here, RNA probes specific to a target gene trigger chain reaction events between two sets of hairpin molecules, allowing for the rapid detection of mRNA (Choi et al., 2014). Multiple different mRNAs can be analysed on a single sample, and the sample can be repeatedly stripped and re-probed. Multiplex HCR has, therefore, significant advantages over more traditional in situ methods. In the complex environment of the developing brain, multiplex HCR has provided an incisive tool to define distinct progenitor subtypes and examine dynamic gene expression patterns. Multiplex HCR can similarly be applied to cultured hypothalamic tissue explants to reveal how naïve or pre-patterned tissues respond to extrinsic signals and/or self-organise (Kim et al., 2022; Chinnaiya et al., 2023). To date, these techniques have provided particularly powerful insights into the development of the tuberal hypothalamus (Chinnaiya et al., 2023).

Here, we describe how to isolate chick hypothalamus at three different stages, culture it as explanted tissue, and process acutely dissected tissue, or explants, by multiplex HCR. The protocols build on previous studies and cover Hamburger-Hamilton (HH) stage 6 to HH stage 25, the time span over which the hypothalamic tissue is induced, regionalised, and begins to undergo neurogenesis. These timepoints equate approximately to E7 and E11.5 of mouse development. We discuss key considerations in deciding how to approach dissection at different stages and steps that aim to minimise technical error and ensure that different samples can be compared. Importantly, the same methodologies can be widely applied to different vertebrates (including rodents), different regions of the brain, and tissues from different germ layers (e.g., Placzek et al., 1993).

## Materials and reagents

**Biological material**

1. Brown Bovan fertilised chicken eggs (Medeggs Ltd)

**Reagents for isolation and culture**

1. Leibovitz’s L-15 medium with phenol red (Fisher Scientific, catalog number: 11415056)
2. Dispase Grade 1 (Roche, catalog number: 4942086001)
3. Corning collagen (Fisher Scientific, catalog number: 11563550)
4. Corning Matrigel GFR membrane matrix (Fisher Scientific, catalog number: 354236)
5. Explant medium:
  Opti-MEM (Thermo Fisher Scientific, catalog number: 31985-062)
  1% L-Glutamine (Fisher Scientific, catalog number: 11574466)
  1% Penicillin/Streptomycin (Fisher Scientific, catalog number: 11528876)
  < 3% Fetal calf serum (Sigma, catalog number: F2442)
6. 0.8 M Sodium bicarbonate (NaHCO_3_), sterile (Sigma, catalog number: S5761)
7. 10× DMEM (without bicarbonate, with phenol red, filtered, sterile) (Thermo Fisher Scientific, catalog number: 31600091)
8. Heat-inactivated goat’s serum (HINGS) (Scientific Laboratory Supplies, catalog number: G9023)
9. Bovine serum albumin (BSA) (Thermo Fisher Scientific, catalog number: B14)
10. Phosphate buffered saline tablets (Fisher Scientific, catalog number: 18912014)
11. Tween 100 (VWR, catalog number: A4974.0250)
12. 70% Ethanol
13. Tissue paper

**Reagents for multiplex hybridisation chain reaction**

1. 0.2 M Phosphate buffer pH 7.4 (PB): 6 g/L Sodium dihydrogen phosphate (NaH_2_PO_4_) (VWR Chemicals, catalog number: 28013.264), 21.8 g/L Di-sodium hydrogen phosphate (Na_2_HPO_4_) (VWR Chemicals, catalog number: 102494C)
2. 4% Paraformaldehyde (PFA) (Sigma, catalog number: P6148) in 0.12 M PB: heat 10 mL of H_2_O to 65 °C, add 1 g of paraformaldehyde, mix by inverting, and add two drops of 1 M NaOH (VWR chemicals, catalog number: 28244.262). Invert until solution clears, make up to 25 mL with 0.2 M PB, and filter
3. Methanol (MeOH) (Fisher Scientific, catalog number: M/4450/17) series in PBS + 0.1% Tween 100 (VWR chemicals, catalog number: A4974.0250) for dehydration and rehydration of explants (25% MeOH/75% PBST; 50% MeOH/50% PBST; 75% MeOH/25% PBST; 100% MeOH)
4. Custom probes (Molecular Instruments)
5. Custom amplifiers (hairpin h1 and hairpin h2) (Molecular Instruments)
6. Hybridisation buffer (Molecular Instruments, catalog number: BPH01825)
7. Wash buffer (Molecular Instruments, catalog number: BPW02025)
8. Amplification buffer (Molecular Instruments, catalog number: BAM012125)
9. Proteinase K (Thermo Fisher Scientific, catalog number: 11444822)
10. Saline-sodium citrate (SSC) (Sigma, catalog number: S/3320/53)
11. SSC + 0.1% Tween 100 (SSCT)
12. DNase I Recombinant RNase-free Sol (ROCHE, catalog number: 4716728001)
13. Formamide (SLS, catalog number: 47670-1L)
14. OCT cryo-embedding matrix (Fisher Scientific UK, catalog number: 12678646)
15. Acetic anhydride (Sigma Aldrich, catalog number: 320102)
16. Triethanolamine (Sigma Aldrich, catalog number: 90279)

**Imaging**

1. Spinning disk confocal microscope (Nikon, model: W1)
2. Microscope (Zeiss, model: Apotome 2)
3. Microscope (Leica, model: MZ16F)
4. 2% Low melting point agarose (Promega, catalog number: V2831)
5. 4’,6-Diamidino-2-Phenylindole, dihydrochloride (DAPI) (Thermo Fisher Scientific, catalog number: D1306)
6. Anti-fade mounting medium (SLS, catalog number: F6182-20ML)
7. Glass-bottomed dishes (VWR, catalog number: 734-2906)

## Equipment

1. Incubators at 18 °C for storing fertilised eggs and at 37 °C for incubating eggs to the right stages (Panasonic, PHCbi Programmable Cooled Incubator 238l)
2. 37 °C, 5% CO_2_ incubators for primary explant cultures
3. Dissection microscope with overhead and under lights (Leica MZ6 Stereomicroscope; Zeiss KL1500 LCD)
4. Dissection tools

- Standard curved forceps (Fine Science Tools, catalog number: 11001-20)
- Hardened fine scissors (Fine Science Tools, catalog number: 14090-11)
- Vannas scissors (Fine Science Tools, catalog number: 15000-01)
- Dumont No. 5 fine forceps (Fine Science Tools, catalog number: 11254-20)
- Micro knives plastic handle (Fine Science Tools, catalog number: 10316-14)
- Tungsten needles (holding and cutting) (Fine Science Tools, catalog numbers: 10130-05 and 20616-12)
  1. Hamburger Hamilton staging guide (Hamburger and Hamilton, 1951)
  2. Nunc 4-well dishes (Sigma, catalog number: 734-1175)
  3. 10 cm, 30 mm Petri dishes (SLS, catalog number: PET2000)
  4. Eppendorfs (VWR, catalog number: 525-0794)
  5. Tissue chopper (McILWAIN Tissue Chopper)
  6. Razor blade (Wilkinson’s) (optional)
  7. Water bath at 65 °C
  8. 3 mL wide-bore Pasteur pipettes (VWR, catalog number: SLIN200C)
  9. Gilson pipettes

## Software and datasets

1. Axiovision software (Zeiss)
2. LAS X1.1.0.12420 imaging software
3. Fiji ImageJ2 Version 2.3.0/1.53q
4. Adobe Photoshop 2023 Version 24.7.0

## Procedure

- **Removal of embryo from egg**
  The following section describes the removal of a chick embryo from the egg. The method is applicable to all patterning stages of development. Isolation can be performed outside a laminar flow hood, but eggs and instruments should be sterilised with 70% ethanol and, between use, instruments should be placed on sterilised tissue paper (autoclaving dissection tools is not necessary).
  1. Incubate embryos in a 37 °C incubator to appropriate HH stage. Use standard fine scissors to window the eggshell and No. 5 fine-tipped forceps to carefully peel away outer and inner shell membranes and vitelline membrane, without damaging the embryo.
  2. Use No. 5 fine-tipped forceps and standard fine scissors to cut around the embryo. Lift out and place in ice cold L-15 medium in a 10 cm Petri dish (Figure 1B).
    **Figure 1.**
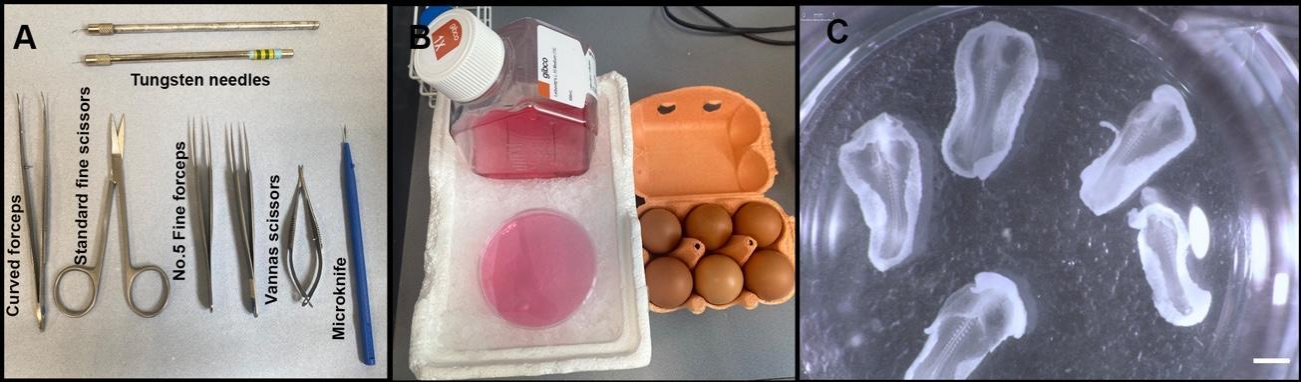
Experimental setup for harvesting chick embryos. (A) Required dissection tools. (B) L-15 medium on ice and Brown Bovan fertilised eggs. (C) Examples of HH10 chick embryos after harvesting from the eggs and removal of membranes, yolk, and excess extra-embryonic tissue. Scale bar: 1 mm.
  3. Remove the chorioallantoic membrane and any contaminating yolk. Transfer embryo (with a minimal amount of medium) to clean L-15 medium in a 30 mm Petri dish using a wide-bore 3 mL Pasteur pipette and assess developmental stage.
  4. Cut away excess extra-embryonic tissue using Vannas scissors or an electrolytically sharpened tungsten needle, to leave the embryo surrounded by a small amount of extra-embryonic tissue (Figure 1C).
  5. If required, analyse a small piece of embryonic tissue by PCR to determine sex. The protocol we describe below is equally applicable to male and female animals.
  **Top tip:** 5 min rule. After removal from the egg, isolate the embryo from extraembryonic tissues as rapidly as possible (within 5 min). Transfer embryo to ice and keep on ice at all times to avoid tissue and RNA degradation.
- **Hypothalamic isolation**
**B1. Method 1**
**Isolation of neuroectoderm**
  The following section describes the isolation of anterior neuroectoderm at three different stages: neural plate [Hamburger-Hamilton (HH6–HH8)], neural tube (HH9–HH12), and phylogenetic (HH13–HH25) stages. The same protocol can be adapted to isolate tissues from other germ layers and other species. Dispase is particularly effective in rapidly and gently separating the neuroectoderm from other tissue layers, while preserving its integrity and viability.
  1. Using tungsten needles, trim embryo in cold L-15 medium to isolate anterior embryonic regions, discarding posterior portions of the embryo and any remaining extraembryonic tissue (Figure 2A, 2F, 2K). Leave sufficient excess tissue to avoid damaging the hypothalamus at subsequent steps in the procedure, so make a posterior cut at the level of Hensen’s node (at HH6), somite 1 (at HH7–HH12), and midbrain (HH13–HH25).
    **Figure 2.**
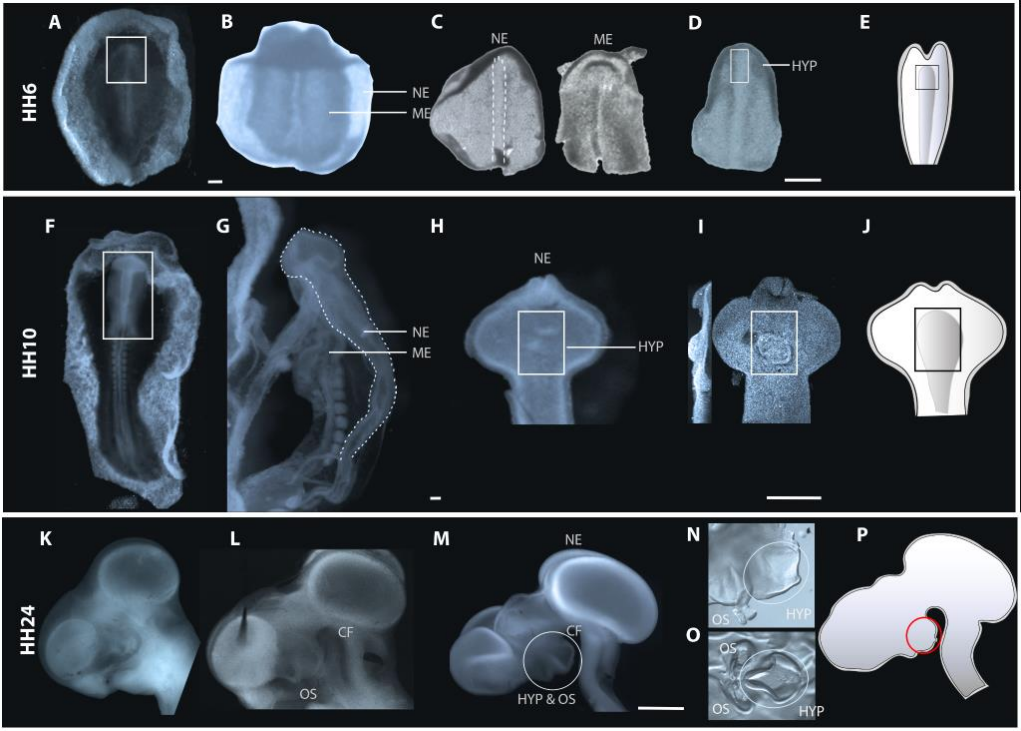
Steps involved in Dispase treatment and sub dissection of hypothalamic tissue at neural plate, neural tube, and phylogenetic stages of development. (A, F, K) Ventral views of whole embryos (A, F) or side view of head (K) harvested from eggs before Dispase treatment. At late phylogenetic stages, the eye is removed prior to Dispase treatment. (B–D) Isolation at neural plate stages. (B) Boxed region in (A) following isolation and Dispase treatment. (C) HH6 neuroectoderm or mesendoderm, separated after Dispase treatment. Dotted outline shows translucent medial midline neuroectoderm cells. (D) Same isolated neuroectoderm as in (C), trimmed to remove head fold and ectoderm. Boxed region shows prospective hypothalamus. (G–I) Isolation at neural tube stages. Note: routinely, the boxed region in (F) would be isolated and treated with Dispase. However, to better show tissue separation, the view in (G) shows an entire HH10 embryo after Dispase treatment. Dotted line in (G) outlines neuroectoderm separating from adjacent mesendoderm. (H, I) Ventral views of isolated anterior neuroectoderm, visualised under the dissecting microscope (H) or brightfield illumination after DAPI labelling (I). Boxed regions show developing hypothalamus, which develops around morphologically distinct neuroepithelial folds. (K–O) Isolation at phylogenetic stages. (L) Side view of HH24 head following Dispase treatment. Optic stalks and hypothalamus start to become morphologically distinct. (M) Isolated neuroectoderm: circled region marks hypothalamus and optic stalks. (N) Side view shows sub-dissected neuroectoderm (circled region in (M). Ellipse outlines prospective hypothalamus which protrudes ventrally between the morphologically distinct optic stalks and the cephalic flexure. (O) Open-book preparation of tissue shown in (N). The violin-shaped hypothalamus is morphologically distinct. (E, J, P) Schematics of HH6, HH10, and HH24 neuroectoderm and highlighted region shows the dissected region of interest. Ellipse outlines hypothalamus. Abbreviations: HYP: hypothalamus; ME: mesendoderm; NE: neuroectoderm; OS: Optic stalk, CF: cephalic flexure. Scale bar: 250 μm.
  2. Transfer the anterior embryonic region into 0.5 mL of freshly made Dispase (1 mg/mL) in L-15 medium in a Nunc 4-well dish at room temperature. Depending on the size of tissue, transfer using siliconised glass pipette, appropriately sized Gilson pipette (P200 or P1000), or forceps (from small to large size, respectively), taking care to transfer minimal amounts of L-15 medium. The length of time of incubation is highly stage dependent (see Table 1). A rule of thumb is to observe the embryos until the tissue layers begin to visibly separate: the mesendoderm shrinks relative to ectoderm (Figure 2B, 2G, 2L). Once the reaction is complete, transfer the embryo regions from Dispase solution into a large volume of cold L-15 medium in a 10 cm dish. Allow embryos/embryonic regions to rest for 5–10 min.
    **Table 1.** Duration of Dispase treatment for the different chick developmental stages

HH stage	Duration of 1 mg/mL Dispase treatment
HH6	5 min
HH8	6–8 min
HH10	10–12 min
HH13/14	15–17 min
HH17–20	18–20 min
HH21–25	20–25 min
  **Top tip 1:** Transferring embryo regions between dishes must be done with care to ensure that they do not become stuck in the pipette or rise and burst at the meniscus [the latter occurs readily if embryos are transferred from a warm to a cold solution, e.g., Dispase solution (room temperature) to cold L-15 medium]. To avoid, transfer under the microscope, avoid air bubbles, and transfer only a few embryo regions at a time (3–4).
  **Top tip 2:** It is essential to optimise the Dispase concentration and incubation period. Tissues exposed to low concentrations of Dispase (even for longer incubation periods) will fail to separate; those exposed to high concentrations of Dispase, or incubated for too long, will become sticky, leading to a failure to obtain cleanly isolated tissue layers.
  **Top tip 3:** For mouse tissues, which are more delicate than chick, reduce time in Dispase by 20%.
  **Top tip 4:** Dispase-treated tissue fragments are transferred with a minimal volume of liquid into a large volume of L-15 medium, negating the need to inactivate the enzyme. However, if necessary, Dispase can be inactivated using a drop of 10% HINGS or BSA.
**Hypothalamus dissection**
  The following section describes the isolation of hypothalamic tissue at neural plate, neural tube, and phylogenetic stages.
  1. Use tungsten needles (some prefer a combination of tungsten needles and micro knives) to dissect the neural tissue (i.e., neural plate, neural tube, or brain, depending on embryonic stage) away from other germ layers. To do so, use a *holding needle* in one hand to pin down the embryo region (avoid pinning down the hypothalamus), and use the long edge of the second needle to gently stroke away the mesendoderm, paring it from adjoining neuroectoderm. The ease and details through which this is achieved is stage dependent. At neural plate stages, the mesendoderm can be easily stroked away from adjacent neuroectoderm. The tissues can be readily distinguished: neuroectoderm is smoother and larger than mesendoderm (Figure 2C). The anterior–posterior axis can be distinguished through the headfold; the neural plate medial midline (which harbours prospective floor plate and hypothalamus) is obvious through its translucent appearance (Figure 2C, dotted outline). At neural plate stages, the neuroectoderm is contiguous with ectoderm, which will provide a useful *holding* tissue at subsequent stages of dissection. At neural tube and phylogenetic stages, mesendoderm and neuroectoderm do not separate as easily, and mesendoderm must be carefully peeled away around the optic vesicles, optic stalk and eyes, and hypothalamus. This can be achieved using tungsten needles and (where tissues are larger) sharp No. 5 forceps. Once isolated, the neural tissue should appear smooth and epithelial-like, with no contamination of round mesenchymal/mesendoderm cells (Figure 2H, 2M). Transfer isolated neuroectoderm into cold L15 medium using a siliconised glass Pasteur pipette or an appropriately sized Gilson pipette (P10, P20), again ensuring that the tissue does not rise to meniscus and bursts.
  2. Once the neural tissue is dissected, sub-dissect the hypothalamus (Figure 2D, 2H, 2I, 2M, 2N) using morphological criteria to define its limits. At HH6, the prospective hypothalamus is situated in and around anterior-most medial midline cells. Like more posterior midline cells, these are translucent but can be distinguished from these because they are slightly wider (Figure 2D). At HH10, the nascent hypothalamus occupies an area centred around a series of neuroepithelial folds in the ventral neuroectoderm. While most obvious after contrast microscopy (e.g., Figure 2I), the folds are morphologically visible under the dissecting microscope (Figure 2H) and provide a precise reference point for hypothalamic position. At later phylogenetic stages, the hypothalamus protrudes ventrally between the morphologically distinct optic chiasm and cephalic flexure (Figure 2L, 2M). Isolation of hypothalamic tissue is achieved in two steps. First, a domain encompassing the optic stalk, hypothalamus, and more dorsal diencephalic tissue is dissected using tungsten needles (limits shown by ellipse in Figure 2M, isolated region shown in Figure 2N). Second, this tissue is prepared as an *open book*. The hypothalamus is obvious as a violin-shaped domain, composed of a translucent medial domain and a thicker surrounding area, situated posterior to the optic stalks/developing optic chiasm (ellipse in Figure 2O).
  **Top tip 1:** 5 min rule. Except for the Dispase step, avoid removing the embryo from ice-cold L-15 medium for more than 5 min. With practice, the neuroectoderm can be isolated from the mesendoderm within 30–60 s (early stages) and 2–3 min (late stages). A good rule of thumb is to dissect one embryo at a time.
  **Top tip 2:** At neural plate stages, proceed to dissect out the hypothalamus (Figure 2C) as rapidly as possible, or midline cells lose their translucent appearance.
  **Top tip 3:** Routinely sharpen the cutting tungsten needle.
  **Top tip 4:** Dissect in a relaxed and calm environment. Remember to breathe and keep your shoulders loose and back straight.
**B2. Alternate slicing method**
  In the previous method, the anterior neural plate/neural tube is first isolated using Dispase, and the hypothalamus (or prospective hypothalamus) is then sub-dissected. Some find this approach difficult and prefer the following alternate method, in which embryos are first sliced into fragments, including a *hypothalamic-containing fragment* that is then treated with Dispase, and hypothalamic tissue isolated. This method is particularly useful for neural tube–stage embryos, where some find it difficult to pare away mesendoderm from the neuroepithelial folds that characterise the nascent hypothalamus at this stage.
  1. Transfer the dissected embryo to a clean 5 cm square plastic plate with as little liquid as possible (it must not dry out but must not float) and arrange it so it is flat (Figure 3A).
    **Figure 3.**
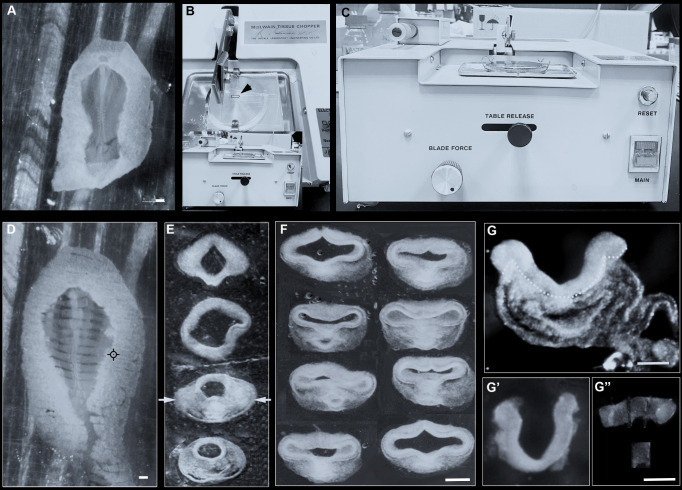
Isolation of hypothalamus tissue using the slicing method. (A) HH9 embryo laid flat on a plastic plate. (B) Top view of tissue chopper, with plastic plate and embryo (dotted outline and arrow) ready for slicing. (C) Front view of tissue chopper. (D) HH9 embryo sliced into 150 μm thick slices. (E) Anterior-most slices, removed from the plastic holder, arranged from anterior (top) to posterior (bottom). Arrows point to hypothalamus-containing slice. (F) Examples of HH10 slices, each containing hypothalamus, after Dispase treatment. (G) Slice containing hypothalamus from a HH9 embryo after Dispase treatment: dotted outline demarcates neuroectoderm and mesendoderm. (G’–G”) Same slice after isolation of neuroectoderm (G’) and sub-dissection to obtain hypothalamic explant (G”). Scale bar: 250 μm.
  2. Set the plate with the embryo onto the base of the tissue chopper, with the anterior–posterior axis of the embryo at a right angle to the tissue chopper blade (Figure 3B).
  3. Set the thickness of each slice by turning the thickness dial at the front (Figure 3C) and use a new razor blade for each experiment to keep the sample sterile.
  4. Move the holder base to the start position and press start.
  5. Once the tissue is sliced (Figure 3D), carefully transfer anterior slices to L-15 medium in a 30 mm Petri dish using a siliconised glass pipette or Gilson (P2 or P10). Each slice has a distinctive morphology. Slices containing the hypothalamus are thicker than more anterior slices, due to the folded neuroepithelium and the underlying mesendoderm, and have a wider neural tube than more posterior slices (Figure 3E).
  6. Transfer hypothalamus slices to Dispase for 5 min (Figure 3F); then, isolate the neural tissue by gently stroking away the adjacent mesendoderm, as above. Once freed from adjacent tissue, the neuroepithelium will constrict slightly (Figure 3G, 3G’), but the ventral midline containing the nascent hypothalamus remains distinct and can be sub-dissected with tungsten needles (Figure 3G”).
  Isolated hypothalamic tissue can be subjected to a wide range of further investigations, ranging from tests of function in vivo (e.g., grafting it to ectopic locations or cell dissociation for scRNA-Seq analyses) to tests of function ex vivo (e.g., neurospherogenic competence or ex vivo cultures). Here, we focus on the investigation of hypothalamic tissue explants in 3D culture systems.
- **Tissue embedding for three-dimensional explant culture**
  Tissue explants are a highly effective tool to investigate the extrinsic signals and tissue-intrinsic self-organising features that orchestrate hypothalamus development. They are versatile and can be used to assay patterning, proliferation, and migration. Explant cultures may consist of the entire hypothalamus or smaller, sub-dissected domains (limited by size). Good viability and the development of explants in a manner analogous to their in vivo counterparts occurs when explants are relatively small. Explants of this size can be cultured for up to seven days, after which tissue crowding and cell death begin to be observed in the explant centre. When used to assay the effects of signalling ligands or inhibitors, these factors can be added along with explant medium. Explants can be embedded in different matrices, most frequently in collagen or Matrigel. Explants are placed on a bottom layer of gelled collagen or Matrigel, then covered with a second layer, and positioned at the interface of the layers. Previous reports have provided detailed methodologies for how to embed explants in 3D matrices (Placzek and Dale, 1999; Placzek, 2008), so here we provide only brief details.
**For collagen:**
  1. Prepare the desired volume (25 μL/bed/well) of 90% collagen and 10% 10× DMEM in an Eppendorf and vortex for 15–20 s. Add 0.8M NaHCO_3_ to make the solution turn pale yellow after vortexing. Typically, 2–6 μL of NaHCO_3_ is added to 100 μL of collagen/10× DMEM. Collagen, if unused, should be left on ice as it will start to set at room temperature. Always make fresh batches of collagen for bottom and top layers.
  2. In a Nunc 4-well dish, prepare collagen beds by pipetting 20–25 μL of collagen, spreading it into a flatbed (Figure 4A). Allow to set (20–30 min at room temperature). Flat collagen beds are easier to embed the tissue as the tissue tends to slide off a convex bed.
    **Figure 4.**
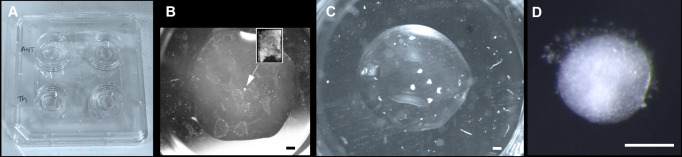
Hypothalamic tissue embedding and culture. (A) Nunc 4-well dishes with Matrigel beds. (B) Single hypothalamic explant after dissection (inset) and embedding (arrow), prior to culturing. (C) Hypothalamic explants cultured after 72 h. (D) High power view of explant cultured for 72 h. Scale bar: (B, C) 250 μm, (D) 100 μm.
  3. Transfer explants onto the collagen bed using a siliconised glass pipette/appropriately sized Gilson (P2, P10, P20), position them, and remove excess medium using a fine pipette (Figure 4B). Overlay with 25 μL of collagen, spreading this to ensure it covers the bottom bed. Reposition/manipulate the explants as required while the collagen is setting. Collagen sets quickly at room temperature so work rapidly and add top collagen layer to one well at a time, providing the opportunity to reposition explants before the collagen begins to set.
  4. Allow the top collagen bed to set completely at room temperature (check by prodding the collagen with a tungsten needle; set collagen feels firm) before adding 400–500 μL of explant medium with or without factors and transfer to incubator for the desired time of incubation (Figure 4C, 4D). Routinely, we culture explants for 3 h to 7 days.
**For Matrigel:**
  1. Prepare desired volume (25 μL/bed/well) of 50% Matrigel and 50% explant medium in an Eppendorf and vortex for 15–20 s. If unused, Matrigel should be left on ice as it will start to set at room temperature. Always make a fresh batch of Matrigel for bottom and top beds.
  2. Proceed with steps 2–4 as above for collagen, but beds must be set by placing dish at 37 °C for a minimum of 30 min (check by prodding Matrigel with a tungsten needle; set Matrigel feels firm).

## Data analysis

**Analysis by multiplex HCR**

**Figure 5.**
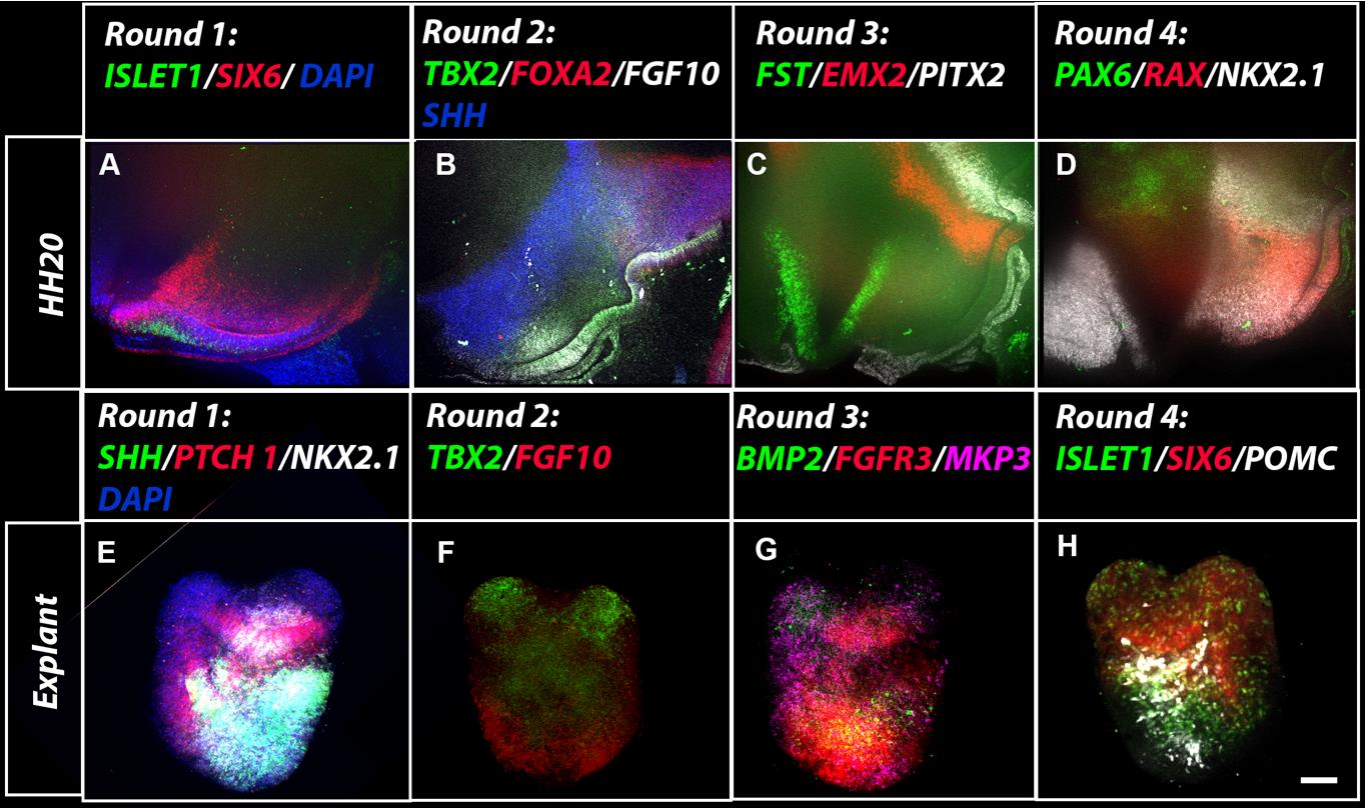
Wholemount multiplex hybridisation chain reaction (HCR) analysis on embryos and explants (A–D) HCR analysis on hemi-dissected HH20. Views show hypothalamic region in four sequential rounds of HCR following stripping and re-probing. Tuberal regions of the hypothalamus express *ISL1, SIX6, SHH*, FGF10, *TBX2, RAX*, and *NKX2-1*; hypothalamic mammillary and supramammillary regions express *FOXA2, PITX2*, and *EMX2. NKX2-1* is additionally expressed in the ventral telencephalon and *PAX6* in the dorsal diencephalon (from (Chinnaiya et al., 2023). (B) Multiplex HCR analyses on wholemount HH6 explants embedded in Matrigel and cultured for 72 h. The explants have been taken through four rounds of HCR following stripping and re-probing. In this example, the explant included prospective tuberal hypothalamus and some eye tissue. Scale bar: 100 μm.

Embryos and cultured explants can be analysed by immunohistochemistry (Manning et al., 2006), flow cytometry (Perez et al. 2023), chromogenic in situ hybridisation (Manning et al., 2006), or multiplex hybridisation chain reaction (HCR). In our hands, we have found that immunohistochemistry and chromogenic in situ work equally well for explants embedded in either collagen or Matrigel, but HCR works well only in explants embedded in Matrigel. Previous reports have described methodologies for multiplexed quantitative HCR (Choi et al., 2020) and for its extension to immunohistochemistry (Schwarzkopf et al., 2021). Here, we extend these reports to provide details on how to take embryos and explants through repeated rounds of multiplex HCR. Such stripping and re-probing enables samples to be routinely analysed with 10–12 genes per sample (Chinnaiya et al., 2023) (Figure 5).

Embryos and explants can each be processed as wholemounts or as cryosections, as detailed below. After completion of the HCR, neuroectoderm can be particularly well visualised either by hemi-dissecting embryos along a sagittal midline (we refer to these as hemiviews), or by isolating the neuroectoderm. Depending on the stage, this can be achieved manually or may require the use of Dispase, as above. Explants are not removed from the Matrigel during the HCR.

**Hybridisation chain reaction**

**Analysis of wholemount embryos or explants**

**Pre-incubation stage**

1. Fix, dehydrate, then rehydrate the samples with a series of Methanol/PBST washes for 5 min each on ice.
2. Incubate samples in Proteinase K (10 μg/mL) for 2–5 min (depending on sample developmental stage) at room temperature.
3. Postfix samples in 4% PFA at room temperature for 20 min.
4. Wash samples with PBST, PBST + 5× SSCT (50:50), and then with 5× SSCT for 5 min each on ice.

**Detection stage**

1. Pre-incubate samples in hybridisation buffer for 30 min at 37 °C.
2. Make up the desired probe solution by adding 2–10 μL of 1 μm stock probe in 500 μL of hybridisation buffer and incubate samples in the probe solution overnight at 37 °C.
3. Next day, remove excess probes by washing samples 4 × 5 min in wash buffer at 37 °C.
4. Wash samples 2 × 5 min with 5× SSCT at RT.

**Amplification stage**

1. Incubate samples in amplification buffer for 5 min at room temperature.
2. Prepare 30 pmol of hairpin h1 and 30 pmol of hairpin h2 in separate tubes by snap-cooling 10 μL of 30 μM stock (heat at 95 °C for 90 s and cool to room temperature in a dark drawer for 30 min).
3. Prepare hairpin mixture by adding snap-cooled h1 and h2 hairpins to 500 μL of amplification buffer at room temperature.
4. Incubate samples in hairpin mixture in the dark overnight at room temperature.
5. Next day, remove hairpins by washing with 5× SSCT at room temperature.
  - 2 × 5 min
  - 2 × 30 min
  - 1 × 5 min
6. Incubate samples in 1:1,000 DAPI (1 mg/mL) for 5–10 min in 5× SSCT.
7. Remove excess DAPI by washing with 5× SSCT.
8. Samples can be stored at 4 °C protected from light before microscopy.

*Note: Leave the samples on a rocker for all the steps except when in Proteinase K.*

**Stripping and re-probing**

1. Following imaging the first round of probes, wash samples 2 × 5 min with 5× SSCT.
2. Pre-incubate samples in 1× DNase buffer (100 μL/mL in H_2_O) solution for 5 min.
3. Incubate samples in DNase1 solution (1:50, 10,000 U/mL) overnight at 37 °C.
*Note: For 500 μL of DNase1 solution: 50 μL of DNase1 + 50 μL of 10× DNase buffer + 400 μL of ddH_2_O.*
4. Check samples under the microscope to confirm that the signal from the previous round is gone.
5. Next day, wash samples 3× 10 min with 30% Formamide in 2× SSCT at 37 °C.
6. Wash samples (3× 10 min) with 2× SSCT at 37 °C.
7. Follow the detection and amplification steps.

*Note: Stripping and re-probing can be repeated successfully for 3–6 rounds with different probe sets.*

**Image acquisition and analysis**

1. Samples taken through HCR analyses can be imaged as flat mounts on a depression slide with anti-fade mounting medium or after embedding in 2% low melting point agarose on a glass-bottomed dish.
2. Samples can be stored at 4 °C protected from light before microscopy.
3. Samples are imaged as z-stacks using Nikon NIS elements or Zeiss Axiovision software and displayed as maximum intensity projections.
4. Image analysis is performed using Fiji ImageJ software.

**Analysis of cryo-sectioned embryos or explants**

For detailed protocol on OCT mounting and cryosection of samples, refer to Manning et al. (2006).

**Pre-incubation stage**

1. Pre warm humidification chamber at 37 °C.
2. Place slides flat on a slide holder and wash with 1× PBS for 5 min to remove any OCT.
3. Post-fix with 4% PFA for 10 min at room temperature. Wash slides 3 × 5 min with PBS.
4. Incubate samples in Proteinase K (10 μg/mL) for 2–5 min (depending on sample developmental stage) at room temperature and fix sections again for 10 min. Wash slides 3 × 5 min in PBS.
5. Treat slides with acetylation mix for 10 min: 11.2 μL of Triethanolamine + 2.5 μL of acetic anhydride in 1 mL of ddH_2_O. Wash slides 3 × 5 min in PBS.

**Detection stage**

1. Pre-incubate slides in hybridisation buffer for 1 h at 37 °C in a humidified chamber.
2. Prepare probe solution as described above in Detection stage.
3. Remove hybridisation solution, apply 200 μL of probe solution to each slide, coverslip the slides, seal the sides of the humidified chamber with Sellotape, and incubate at 37 °C overnight.
4. Next day, immerse slides in probe wash buffer at 37 °C to float off the coverslip.
5. Remove excess probe by incubating slides at 37 °C in:
  - 75% of probe wash buffer/25% 5× SSCT for 15 min.
  - 50% of probe wash buffer/50% 5× SSCT for 15 min.
  - 25% of probe wash buffer/75% 5× SSCT for 15 min.
  - 100% 5× SSCT for 15 min.
6. Wash slides in 5× SSCT for 5 min at room temperature.
7. Dry slides by blotting edges on tissue paper.

**Amplification stage**

1. Add 200 μL of amplification buffer and pre-amplify for 30 min at room temperature.
2. Prepare hairpin mixture as described above and add 200 μL of hairpin mixture, coverslip, and incubate overnight at room temperature.
3. Next day, immerse slides in 5× SSCT to float off the coverslip.
4. Remove excess hairpins by washing with 5× SSCT at RT for:
  - 1× 5 min.
  - 2× 30 min.
  - 1× 5 min with DAPI.
  - 1× 5 min.
5. Dry slides by blotting edges on tissue paper.
6. Add 50–100 μL of anti-fade mounting medium and mount the coverslip onto the slide for microscopy.

**Stripping and re-probing**

1. Following imaging the first round of probes, wash samples 2× 5 min with 5× SSCT.
2. Pre-incubate in 1× DNase buffer (100 μL/mL in H_2_O) solution for 5 min.
3. Incubate samples in DNase1 solution (1:20, 1,000 U/mL) for 4 h at 37 °C.
*Note: For 500 μL of DNase1 solution: 25 μL of DNase1 +50 μL of 10× DNase buffer + 425 μL of ddH_2_O.*
4. Check slides under the microscope to confirm that the signal from the previous round is gone.
5. Next day, wash samples 3× 10 min with 30% Formamide in 2× SSCT at 37 °C.
6. Wash 3 × 10 min wash with 2× SSCT at 37 °C.
7. Follow the detection and amplification steps.

*Note: Stripping and re-probing can be repeated successfully to 3–6 rounds with different probe sets.*

**Image acquisition and analysis**

1. Samples taken through HCR analyses can be mounted with anti-fade mounting medium and coverslipped.
2. Slides can be stored at 4 °C protected from light before microscopy.
3. Samples are imaged as single stack or z-stacks with Zeiss Axiovision software and displayed as maximum intensity projections.
4. Image analysis is performed using Fiji ImageJ software.
  **Top tip 1:** In some cases, morphological criteria can be used to align images from different rounds. When this is not possible, a reference probe can be re-deployed in each round.

## Validation of protocol

This protocol or parts of it has been used and validated in the following research article(s):

- Chinnaiya et al. (2023). A neuroepithelial wave of BMP signalling drives anteroposterior specification of the tuberal hypothalamus. *eLife* (Figure 1, panel T-T’’; Figure 3; Figure 4; Figure 5 panel E–H; Figure 6.
  The robustness and reproducibility of our protocol can be evidenced from the above research article.
